#  A New Era of Prostate Cancer Precision Medicine

**DOI:** 10.3389/fonc.2019.01263

**Published:** 2019-11-26

**Authors:** Adil Malik, Srilakshmi Srinivasan, Jyotsna Batra

**Affiliations:** ^1^School of Biomedical Sciences, Queensland University of Technology, Institute of Health and Biomedical Innovation, Brisbane, QLD, Australia; ^2^Australian Prostate Cancer Research Centre–Queensland, Translational Research Institute, Woolloongabba, QLD, Australia

**Keywords:** prostate cancer, precision medicine, gene fusion, biomarkers, proteomic technologies, genome editing, non-coding genome, liquid biopsy

## Abstract

Prostate cancer is the second most common male cancer affecting Western society. Despite substantial advances in the exploration of prostate cancer biomarkers and treatment strategies, men are over diagnosed with inert prostate cancer, while there is also a substantial mortality from the invasive disease. Precision medicine is the management of treatment profiles across different cancers predicting therapies for individual cancer patients. With strategies including individual genomic profiling and targeting specific cancer pathways, precision medicine for prostate cancer has the potential to impose changes in clinical practices. Some of the recent advances in prostate cancer precision medicine comprise targeting gene fusions, genome editing tools, non-coding RNA biomarkers, and the promise of liquid tumor profiling. In this review, we will discuss these recent scientific advances to scale up these approaches and endeavors to overcome clinical barriers for prostate cancer precision medicine.

## Introduction

Prostate cancer (PCa) is the second commonly diagnosed cancer (after skin cancer) in the Western society. In 2018, worldwide there were 1.3 million new patients diagnosed with PCa (1). The usage of biomarkers for PCa screening, detection, and prognosis have transformed the diagnosis and management of the disease. Introduction of prostate-specific antigen (PSA) test in clinical practice has ensured early identification and decreased mortality from PCa (2). In spite of its ample value for PCa detection, PSA as a solitary test has some restrictions. The PSA test has specificity and sensitivity ranging from 20 to 40% and 70 to 90%, respectively, reliant on the applied cutoff for PSA levels (PSA > 4 ng/ml as normal) (3). One explanation for the poor specificity of the PSA blood test is that some non-cancerous causes may escalate the PSA level in men. For example, benign prostate hyperplasia (BPH) and prostatitis may cause an elevation in PSA levels, and there is little evidence to indicate that BPH and prostatitis will develop into PCa (4). Scientists and clinicians are still debating on the use of a single PSA test vs. standard non-screening practices on PCa mortality. A recent study called as cluster randomized trial of PSA testing for PCa (CAP) was conducted in the UK including 419,582 men in 50–69 age range (5). The results indicated no significant difference in PCa mortality of men screened for PSA vs. non-screened patients after a median follow-up of 10 years, but increased early stage low-risk PCa detection. The diagnostic approaches for detecting PCa are changing with advancement in imaging and biomarker discovery to improve early-stage PCa detection. Consequently, despite substantial advances in the exploration of PCa biomarkers, few men are overdiagnosed with inert PCa, while others are missed, developing the invasive disease and diagnosed at a late stage (2).

Several management options are available for patients ranging from active surveillance for less aggressive PCa to surgery and radiation for advanced disease. Early studies on androgen deprivation in PCa demonstrated the role of androgen receptor in growth and survival (6). Androgen deprivation therapy (ADT) is presently the primary antihormone therapy for treating advanced PCa. Apart from the initial efficacy of ADT, most patients with advanced PCa eventually develop resistance to this therapy and progress to castrate-resistant PCa (CRPC) (7). Antiandrogen drugs such as enzalutamide, abiraterone, and apalutamide have been Food and Drug Administration (FDA)-approved second generation therapies, which has increased survival of CRPC patients (metastatic and localized) (8, 9). Alternatively, chemotherapy presents a viable option for treating metastatic PCa and has appeared to increase survival rate compared to ADT (10). The standard chemotherapy agent, docetaxel, has been used primarily until cabazitaxel was approved in 2010 for CRPC patients (11). In spite of these durable therapies, majority of the cases do not respond to initial therapy due to adaptive resistance, induction of immunosuppressive pathways in the tumor, resulting in tumor relapse (12). An FDA-approved cancer cell vaccine, sipuleucel-T, has been slightly beneficial for increased survival for patients with advanced PCa (13). Several immunotherapy clinical trials for patients with advanced PCa are still underway and awaiting final outcomes.

Multiple studies have discussed the role of clinically relevant mutations, as well as the level of tumor heterogeneity in primary prostate tumors (14). Genetic tools developed in this “genetic revolutionary” era have been useful to understand this heterogeneity in PCa tumors and identify the finest treatment for patients; new technologies like genomic profiling and use of poly-(adenosine diphosphate) [ADP]-ribose polymerase (PARP) inhibitors like olaparib for patients with mutations in DNA damage response genes have been a boon for precise and effective extrapolation of therapies for individual cancer patients (15). A phase II study by Mateo et al. in CRPC patients, described an increased response of PARP inhibitors to patients with mutations (somatic and germline) in DNA repair genes (16). Improved technologies to interrogate cancer genome have found somatic and germline associations with cancer risk identifying alterations and targets in defined genomic subset of patients. For instance, aberrations in androgen receptor (*AR*), *TP53*, retinoblastoma 1 (*Rb1*), *BRCA*1, and *BRCA2* genes have been evaluated in PCa (17). Somatic mutations in DNA repair genes including *BRCA1* and *BRCA2* have been reported in PCa patients; wherein *BRCA2* mutations (12%) were found to be more frequent compared to *BRCA1* (2%) in advanced PCa patients (18). Castro et al. evaluated the status of *BRCA1/2* in 2,019 patients diagnosed with PCa. They confirmed the presence of *BRCA* mutations in aggressive phenotype, with poor survival outcomes (19). The same group investigated the influence of *BRCA* mutations in treatment outcomes in a cohort of 1,302 PCa patients including 67 *BRCA* mutation carriers. The results indicated that *BRCA* carrier patients undergoing radiotherapy or prostatectomy had shorter survival and developed metastasis sooner compared to non-carriers (20). A recent study identified a germline BRCA2 mutation (c.4211C > G) in a Chinese patient treated with ADT and radiotherapy, the mutation resulting in a truncated protein. The researchers demonstrated that PCa associated with this mutation is sensitive to ADT + radiotherapy and may be effective in patients with this mutation (21).

**Figure 1:**
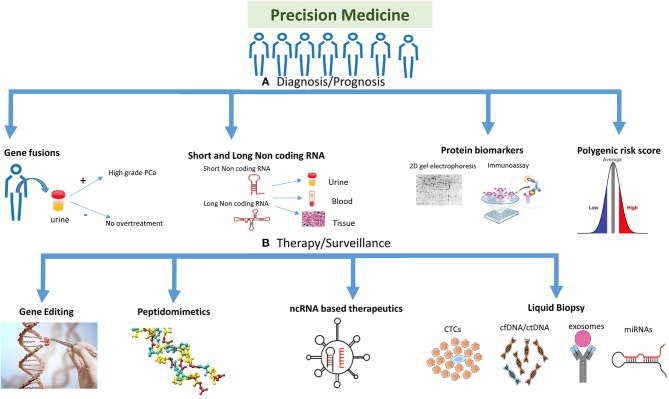
Highlighting the different strategies used for precision medicine. The precision medicine approach could be divided into different strategies and technologies which are being used to target the disease. **(A)** Diagnosis/prognosis: polygenic risk profiling could help differentiate a population or individual into high/intermediate/low risk patient, whereas molecular markers like gene fusions, protein biomarkers (e.g., 2D gel electrophoresis, MS-based proteomics and immunoassays) and non-coding RNA (short and long) could help detect prostate cancer (PCa) at different stages of the disease including primary tumor stage or treatment response. Gene fusions could help in detecting PCa at different stages and also in reducing overtreatment for patients. **(B)** Therapy/surveillance: clinical utility of circulating tumor cells (CTCs), circulating tumor DNA (ctDNA), and cell free DNA (cfDNA), microRNAs (miRNAs), and exosomes represents an evolution in cancer diagnosis, prognosis, and treatment. New viral/nanoparticle-based non-coding RNA (ncRNA) therapeutics have evolved in the twenty-first century with many siRNA and miRNA-based therapies in clinical trials. Antisense oligonucleotides and peptidomimetics offer an out-of-the-box approach to target genes and proteins at transcriptional and translational levels repressing their activities. Gene editing is a fascinating approach being improved on a daily basis, which could target the disease at DNA level to repair mutations or inhibiting fusion genes. Gene editing image credit: Getty images (https://bit.ly/2ql54Gk).

As the one-size-fits-all approach used in traditional medicine to treat PCa has failed to benefit the patients, the need of the hour is to develop the precision medicine approach which would help patients in the long run. New genomic and proteomic technologies, gene editing technologies, non-coding RNA diagnostics and therapeutics, and liquid tumor profiling have the potential to captivate the promise of precision medicine, highlighting this revolution on different aspects of cancer and their translatability into clinics (Figure 1). In this review, we discuss about the emerging technologies and tools for PCa precision medicine.

## Genomics And Fusions in PCa Precision Medicine

Genetic influences on PCa have been well-recognized, and our understanding of the molecular genetics of the disease is improving (22). Genetic predisposition could play a decisive role in determining whether a patient should undergo screening and also predict the stage at which the screening may be performed. Early detection of disease and prevention are primary goals for an advancing scientific research community. Genome-wide association studies (GWAS) have been useful in determining genetic risk variants associated with PCa. GWAS involves the investigation of at least hundreds of thousands of variants throughout the genome in large cohorts of individuals, often split into cases and controls, to recognize variants associated with the trait of interest. The most common types of variations in the human genome are termed single nucleotide polymorphisms (SNPs) and are believed to directly contribute to the progression of many complex diseases, including PCa (23).

Numerous advances in high-throughput genotyping have improved the performance of GWAS and even more recently detailed whole-exome and whole-genome sequencing studies. Currently, more than 150 loci were reported to be associated with PCa susceptibility and aggressiveness that accounts for ~40% of PCa risk (21, 24). Risk information could be collected and analyzed using an array of SNPs and estimate an individual's risk of developing a disease (25). This risk is calculated by sum of all the risk SNPs corresponding to a phenotype, to the effects of GWAS on the same phenotype. This prediction of polygenic risk could improve clinical decisions in screening for PCa (26). Using this model of polygenic risk score (PRS)/polygenic hazard score (PHS), men at high cancer risk could be identified thus, reducing morbidity and mortality. Recent studies have suggested that polygenic risk profiling on the basis of PRS could provide personal and clinical utility to patients and health management (27, 28). These risk variants have been used to determine a PRS estimating an individual's risk to developing disease (29, 30). In a recently published research study, Seibert et al. calculated PHS for age-related risk from SNPs, which predicted diagnosis of aggressive cancer (31). The study used data from 31,474 men of European ancestry, which had a total of 201,043 SNPs for analysis. In a validation set of 82,429 men, 54 SNPs predicted a higher score for aggressive cancer. Moreover, increased PHS led to a higher prediction for aggressive PCa with PSA screening.

PSA screening test has led to high false positives with overtreatment of benign disease. PRS has been observed to aid in identifying men with elevated PCa risk attaining greater benefit/risk ratio compared to PSA testing (31, 32). Men in the top 50% with PRS scores account for 76% of PCa with the top 20% individuals accounting for 42% of aggressive PCa (31). PRS does not directly correspond to aggressive PCa but solves the false-positive PSA screening and associated overtreatment of disease (33). Additional clinical risk factors with well-developed active surveillance may help decide if treatment is needed for PCa (34). This PRS-regulated PCa screening would lead to individuals being recommended screening at specific age. A recent investigation by Schumacher and colleagues identified 63 novel variants associated with PCa susceptibility, with the authors claiming that the findings could improve risk prediction using PRS risk assessment with informed screening and help disease management (35). A team led by Lecarpentier et al. suggested that PRS score can be useful determinant for the management of breast or PCa risk in BRCA1/2 mutation carrier men. At 80 years of age at the 5th and 95th percentiles of the PRS varied from 7 to 26% for carriers of BRCA1 mutations and from 19 to 61% for carriers of BRCA2 mutations, respectively. They evaluated prostate and breast cancer risk using PRS based on 88 female breast cancer and 103 PCa susceptibility variants (36). Although this study gives an improved screening strategy for mutation carriers in whom PRS prediction can be used for reducing over diagnosis in general population, it lacks in assessing the effect of family history with mutation carriers associated with PRS. The core philosophy of precision medicine is personalized management of disease. Recent studies in coronary heart disease have invoked the potential of PRS in therapeutic interventions, with the use of proper dosage of statins in individuals who have at least one risk factor for cardiovascular problem (26). Genetic risk scoring has shown promise in identifying individuals who may profit from early preventive actions and disease management. However, uncertainty looms large on the risk estimates of individuals screened using PRS vs. familial disease trait, with its inability to calculate variants not related to causal genetic factors leading to apprehensions in estimation of risk (37). The discrepancy in using this risk score in different populations is also a major concern to transfer it to clinical settings (38). This risk score bias needs to be validated in a diverse population to remove the effects of demography. These precincts should be looked upon by researchers with developing new methods to improve generalization of PRS. New statistical models to identify and characterize allele frequency in unidentified locus associated with disease would help in calculating genetic risk of an individual with more precision.

Gene fusion is a phenomenon wherein a hybrid gene is formed from two genes by chromosomal inversion, translocation, or deletions (39). Emerging experimental data indicate that gene fusions are important molecular events in the growth and advancement of PCa (40). The most common gene fusions in PCa genomes are with members of the *ETS* family of transcription factors, such as *ERG* and *ETV* genes (41). Assessing ETS status, and developing assays, therefore could probably be a way forward. Immunohistochemistry (IHC) has been a standardized tool to evaluate ETS status in clinical settings; however, this technique falls short of detecting fusions involving other ETS family members. To overcome this problem of characterizing prostate tumor ETS status in clinical samples, Tomlins et al. developed a method, clinical laboratory improvement amendments decipher assay (42). The assay achieved 91% sensitivity and 98% specificity in detecting ETS status when validated in 252 primary prostate tumors and independent 155 radical prostatectomy samples. Torres and colleagues further evaluated this assay to assess ETS status in the Decipher Genomics Resource Information Database transcriptome database, expanding the validation by including *ETV1, ETV4, ETV5*, and *ERG* (43). The model showed 100% robustness among samples for ETS status, with *ETV1, ETV4*, and *ETV5* showing area under the curve (AUC) of 98, 88, and 99, respectively. This approach emerges as an individualized tool for molecular classification of PCa tumors utilizing ETS gene fusion status. This model, when applied to radical prostatectomy samples, predicted m-ERG model sensitivity at 93%. However, in uncommon cases of ETV5 overexpression, this model had a lower sensitivity. In spite of the advantages of this assay, the presence of ETS rearrangements was not associated with adverse outcomes in one of the cohorts used by the group contrasting its previously published data (44). Henceforth, the significance of this data seems unclear at the moment, but the authors argued about the use of this classifier assay as a tool for molecular classifying prostate tumors.

The most common fusions studied in PCa are in ERG, which belongs to *ETS* family. The early twenty-first century saw the discovery of genomic rearrangements in *TMPRSS2* and *ERG* oncogenes in PCa. Later in the decade, it was identified and accepted that ~50% of the tumors in prostate harbored *TMPRSS2–ERG* fusions as the most recurrent genomic modification (45). These rearrangements have been observed in early PCa representing high Gleason Score with poor prognosis as a result of ERG-modulated transcription events affecting PCa cells invasiveness, migration, and epithelial-to-mesenchymal transition (EMT) (46). These fusion genes were detected previously using fluorescence *in situ* hybridization (FISH) or the traditional PCR techniques. Several new *in silico* tools including SOAPfuse, FusionMap, JAFFA, FusionSeq, and GFusion have been developed to identify novel fusion genes using RNA-seq data. These computational tools have presented an alternative platform compared to traditional methods like reverse transcription PCR and FISH to detect fusions with higher sensitivity and a lower false-positive rate (47). It has been suggested that *ERG*-targeted drug development in combination with urine tests available to detect *TMPRSS2–ERG* fusion could define a precision medicine procedure for PCa patients (48, 49). A clinical trial conducted by Jewish General Hospital, Quebec, Canada, recruited 65 high-risk PCa patients treated with radiation and hormonal therapy after biochemical failure to evaluate the predictive value of *TMPRSS2–ERG* gene fusion (ClinicalTrials.gov Identifier: NCT02588404). This study once completed will correlate Gleason Score and T-stage with *TMPRSS2–ERG* gene fusion event. In addition, researchers have also looked at targeting ERG directly; ERG knockdown in VCaP CRPC cell line indicated the role of ERG in proliferation and blocking differentiation of prostate cells to neuroendocrine and luminal cell types (50). These findings by Mounir et al. supported the clinical utility to target these alterations.

A potential therapeutic strategy involving inhibition of ETS cofactors including DNA-dependent protein kinase, PARP1, and histone deacetylase 1 rather than the fusion has generated a lot more interest in the scientific community (51). A randomized phase I and II studies inhibiting PARP1 in CRPC patients were started in 2012 (ClinicalTrials.gov Identifier: NCT01576172). Primary results showed that the addition of veliparib (PARP inhibitor) to abiraterone acetate plus prednisone therapy did not show any response (52). Similarly, phase II trials targeting HDAC in CRPC patients have been completed with disappointing results (53, 54). These results do show that targeting transcription factors does need a novel strategy. A lack of “new-age” compounds targeting ETS factors makes it a tedious task to target these oncoproteins for developing new therapeutics for PCa. There have been advancements in developing small molecule antagonists to ERG; YK-4-279 was discovered as a small molecule targeting FLI1 protein, a homolog of ERG. Its derivative, TK216 is currently in phase I trial and is anticipated to have considerable effect on developing ETS-targeted therapies (55). Computer-aided drug discovery approach has yielded a small molecule compound VPC-18005 that directly targets ERG at low concentrations suppressing metastasis of ERG-expressing PCa cells (56). Wang et al. devised a new approach for targeting oncogenicity of ERG using inhibitory peptidomimetics. The group screened a phage display peptide library, identifying peptides that specifically bind to ERG protein but not to a negative control protein. These ERG-inhibitory peptides (EIP) disrupted ERG-ETS domain/DNA interactions via binding to ETS domain of ERG (57). The observations in this research wherein EIP destabilizes ERG may provide an alternative approach targeting transcription factor for therapeutic purposes. Peptidomimetics offers target specificity with low cytotoxicity and side effects, but vital questions like high developmental costs, delivery, and permeability of the membrane needs to be answered before clinically developing peptides for PCa treatment. While this complex strategy offers specificity and efficient target affinity, issues still remain about solubility, stability, delivery systems, and high cost of development. Future studies should concentrate on overcoming these limitations and developing new inhibitors of ERG to treat resistant and metastatic form of PCa.

In addition to TMPRSS2–ERG/ETS fusion in PCa, new fusion transcripts have been identified in both normal and tumor prostate tissue (58). Zhao et al. identified 21 fusion transcripts that are involved in PCa, out of which 13 were novel fusion transcripts (59). These included fusion transcripts between protein coding genes in addition to long intergenic non-coding RNA (lincRNA) as fusion partners in some transcripts. The fusion transcripts *ACSS1-APMAP, RP11_17A19.1-KCTD1*, and *ZNF841-ZNF432* were found to be highly expressed in tumors vs. benign tissues. The authors found lincRNA fusion to be the most common in PCa, highlighting the biological importance of lincRNA fusion transcripts in tumorigenesis (59). Similarly, Qin et al. found *SLC45A3-ELK4* fusion RNA in PCa cell lines, functioning as a chimeric lincRNA regulating cell proliferation (60). At this point of time, this is a new mechanism to understand the biology of lincRNA fusions in PCa. However, if this mechanism can be elucidated, silencing of these genes using clustered regularly interspaced short palindromic repeat (CRISPR)–CRISPR-associated (Cas) can be achieved. It still remains to be seen how effective are these lincRNA fusions in detecting PCa or targeting them as a therapy.

Lai et al. investigated the mechanisms of fusion gene transcription/splicing from RNA-seq data of prostate tumors and cells using a program FusionMap (61). It was an interesting computational analysis wherein the authors indicated a concurrence of GT-AG intron donor–acceptor splice site in 76% of fusion junctions. In addition, the prediction indicated that non-fusion splice sites and fusion junctions have similarity in hybridization. Some new fusion transcripts were detected in androgen-/antiandrogen-treated cells highlighting the importance of fusion genes in PCa. *C1QTNF3-AMACR* fusion transcript was found to have an expression profile distinct from their parental genes in prostate tumors. Out of 185 newly identified fusion transcripts, some fusion genes including *SIDT2-TAGLN, HARS-ZMAT2, CTBS-GNG5, NOS1AP-c10rf226*, and *DHRS1-RABGGTA* were differentially expressed in prostate tumors as detected in clinical RNA-seq dataset. This uniqueness of cancer driver gene fusions in tumor cells highlight their importance for therapeutic application targeting cancer cells.

Phosphatase and tensin homolog (*PTEN*) is a tumor suppressor gene associated as a genomic marker in various oncological settings. Aberrations in *PTEN* are found to be most common in PCa wherein inactivation and deletion have been identified in prostate tumor patients at radical prostatectomy stage and in CRPC (62). PTEN loss has been positively correlated with the *TMPRSS2:ERG* fusion event, implying roles of both these somatic events in prostate tumorigenesis (63). Clinical studies indicate the association of patient's *PTEN* status with effective therapies. Robust clinical assays like IHC and FISH have enabled clinicians to use PTEN as a prognostic marker in tissue and liquid biopsies (64). PTEN loss has been correlated with increased activation of phosphoinositide 3-kinase–RAC-alpha serine/threonine-protein kinase pathway, which is concomitant with adverse clinical outcomes. These pathways are being tested clinically with new therapeutic compounds in metastatic PCa patients (64). Inactivation or loss of *PTEN* genes leads to PI3K pathway activation leading to tumorigenesis. Recently, Mateo et al. tested a drug, GSK2636771, for the selective pathway inhibition of PI3Kβ (65). The group evaluated different parameters including antitumor activity and the pharmacokinetic and pharmacodynamic properties of this drug and concluded clinical benefit of GSK2636771 as a selective inhibitor of PI3K pathway in patients with solid tumors.

All the studies mentioned in this section are still trying to develop and target these fusion genes; some are currently in development. The status of these fusion genes can be a guidance during therapy surveillance, risk stratification, or as effective target therapies. The detection of this genes and their fusion partners may be effective in prediction of hormonal treatments and classify them as androgen dependent or independent, which could alter therapy stages and improve outcomes. The new approach of peptidomimetics to target these genetic anomalies could be the answer to effectively reduce tumor growth without changing the normal cellular functions. However, before this can be used as precision therapies, modifications to change the peptides needs to be done for proper intake into the cells and inhibit these fusions.

## Protein Biomarkers And New Proteomic Technologies

Proteomic technologies and proteomics have always been a fascinating area of research for the discovery of cancer-specific biomarkers, improvement in prognosis, and treatment response biomarker identification, contributing to in-depth understanding of cancer pathology and developing new effective therapies (66). Mass spectrometry (MS) has been the forefront for proteomic and peptidomic analysis, identifying thousands of proteins and translating relevant data to clinics. In the past decade, different MS-based platforms like liquid chromatography-tandem MS (LC-MS), two-dimensional fluorescence difference gel electrophoresis (2D-DIGE), matrix-assisted laser desorption ionization (MALDI)–time of flight MS, capillary electrophoresis–MS, selected reaction monitoring (SRM)/multiple reaction monitoring (MRM), and parallel reaction monitoring (PRM) have been developed for biomarker discovery, paving the way for translating it to clinically relevant biomarkers in prognosis and diagnosis.

A number of relevant biomarkers (Table 1) and multimarker assessment tests are now available for clinicians to make the difficult clinical decision of treating PCa. Prostate Health Index is a blood test for PCa detection, which involves combining total PSA, free PSA, and proPSA. This test was seen to be discrete and surpass its singular components with additional aspects of predicting progression of PCa during active surveillance (67). Alternatively, PROSTARIX™ is a commercially available urinary test by Metabolon Inc. This test measures a panel of four metabolites (sarcosine, glutamate, alanine, and glycine) by chromatography and MS after digital rectal examination (DRE) (68). This test showed increased specificity and sensitivity vs. serum PSA (AUC 0.78). Another test named ConfirmMDx, an epigenetic test for predicting prostate biopsy using prostate core samples, helps patients in managing PCa at initial diagnosis. A patient is informed about the need for rebiopsy using this test with a sensitivity of 62–68% and a specificity of 64% (69). The Matloc study confirmed the usage of this test to reduce the need for biopsy in many patients with a negative predictive value of 88–90% (70).

**Table 1:** Different studies highlighting the role of proteomic techniques in precision medicine.

**Method**	**Study**	**Sample**	**References**
Quantitative proteomics	Prediction of disease aggressiveness via proteomic biomarkers	Formalin-fixed paraffin-embedded tissue	(71)
MALDI-MS profiling	Potential of β microseminoprotein combined with PSA as biomarkers	Post-DRE urine samples	(74)
2D gel electrophoresis + MALDI-MS	Urinary protein changes after radical prostatectomy	Urine samples postradical prostatectomy	(78)
2D-DIGE/MS	Panel of diagnostic biomarkers (α-1-microglobulin, transferrin, and haptoglobin)	Urine samples	(79)
iTRAQ	Multiplex biomarker panel for diagnosis	Serum and urine	(80)
iTRAQ + SRM/MRM	Proteomic analysis of urinary extracellular vesicles from high grade PCa	Urinary extracellular vesicles	(81)
ELISA + Western blotting	Immunoassay-based validation of urinary exosomal proteins as PCa biomarkers	Exosomes in urine	(83)
IHC + SRM + PRM	Verification of urinary biomarkers using targeted proteomics	Urine	(84)

Other tests have been developed for identifying aggressive disease that can reduce prostate biopsies. An eight-biomarker signature [pS6 (phosphorylated S6), HSPA9, DERL1, PDSS2, YBOX1, CUL2, FUS, SMAD4] tissue-based proteomic assay was developed by Metamark genetics for formalin-fixed tissue biopsies for precision in clinical decision after biopsy. This assay using proteomics looks promising, but efficacy and reproducibility using formalin-fixed paraffin-embedded tissues vs. fresh tissues need further validation (71, 72). Many research groups worldwide are adopting different protein profiling approaches for developing new assays for PCa diagnosis and prognosis (73, 74). Flatley et al. collected pre- and post-DRE urine samples from PCa and other prostatic disease patients and performed MALDI-MS profiling. This approach found evidence of β-microseminoprotein (MSMB) to be a potential biomarker in PCa diagnosis. When combined with serum PSA levels, the sensitivity of MSMB/PSA model gave increased sensitivity of 96% at 26% specificity (74). This model is still insufficient for population screening due to low specificity; however, including MSMB in a large panel of biomarkers would justify its inclusion and reduce the number of needless biopsies in benign patients.

A research was conducted in 2015 called The Stockholm (STHLM3) study to improve the performance of detecting PCa, wherein a combination of genetic tumor markers (232 SNPs), PSA concentration, plasma proteomic markers (MIC-1, MSMB, intact-PSA, free-PSA, and HK2), and standard clinical variables (prostate volume, family history, age, DRE, and previous biopsies) were tested in a cohort of Swedish men aged 50–69 years. The primary aim of the STHLM3 study was to assess the efficacy of this model to increase specificity for detecting PCa. An improved AUC (0.74) compared to standard PSA (0.56) was observed identifying tumors with the STHLM3 model. All the variables used in the study were found to be significantly associated with Gleason Score of at least 7 in multiple logistic regression model. This approach also showed a decrease in benign biopsies by 44% (75). This model clearly outperforms the PSA as a screening tool for PCa. The STHLM3 model was updated wherein intact PSA has been removed and a new biomarker, the rare germline mutation (G84E) in *HOXB13*, has been added to improve this test (76) (ClinicalTrials.gov identifier: NCT03639649). It was found that using this updated test, biopsies were reduced by 34% compared to using PSA alone. This test has also been validated in an independent clinical setting in Norway and Sweden, which showed its efficacy in reducing biopsies in a substantial amount (77). In addition, studies validating this test in non-Caucasian population will commence shortly. Although this test has been found to be clinically better than PSA, there is yet no evidence of it being beneficial to men above 70 and below 50 years old. At this juncture, the STHLM3 test still seems to be the best possible option for patients in the 50- to 69-year-old category.

Owing to the complexity of PCa tumor and its heterogeneity, a signature or a panel of biomarkers could be a more practical and plausible route to the diagnosis and prognosis of this cancer. Three unique proteins, namely, cyclin-dependent kinase 6, Galectin-3-binding protein, and l-lactate dehydrogenase C chain, were identified in urine samples using two-dimensional gel electrophoresis coupled with MALDI–time of flight MS related to surgical margin status after radical prostatectomy. These proteins may assist in evaluating tumor progression after surgical treatment (78). This research on PCa urinary proteome identified differences in positive and negative surgical margin patients after radical prostatectomy, highlighting the importance of combining genomic and proteomic approaches to understand PCa status in patients. Davalieva et al. accounted for the potential of three biomarkers (α-1-microglobulin/bikunin, transferrin, and haptoglobin) in urine using 2D-DIGE/MS. All three proteins yielded different specificity and sensitivity, but the integration of haptoglobin and α-1-microglobulin/bikunin had higher accuracy in PCa detection compared to PSA underlying its potential as a urine based biomarker (79). Quantitative proteomic (MS-based) approaches like isotope-coded affinity tag and isobaric tag for relative and absolute quantitation (iTRAQ), which are isobaric labeling methods, have been used recently without the need of traditional gel electrophoresis methods. Using iTRAQ, Zhang et al. identified three proteins, namely, serum platelet factor 4 variant 1, urinary cysteine-rich secretory protein 3, and PSA for precise diagnosis of PCa. The AUC of this three panel of proteins was higher (AUC, 0.941) compared with PSA alone (AUC, 0.757) for PCa prediction. Platelet factor 4 variant 1 and cysteine-rich secretory protein 3 when combined could attain better discrimination in gray zone of PSA (4–10 ng/ml) and had the potential to differentiate between high-grade prostatic intraepithelial neoplasia and PCa (80). Fujita et al. found five proteins when analyzing urinary extracellular vesicles using iTRAQ and LC-MS/MS to be better predictors of PCa compared to PSA from high Gleason score PCa (81). iTRAQ coupled with 2D-LC-MS/MS was used by Katafigioti et al. and team for the proteomic profiling of PCa tissue. They detected potential protein biomarkers in PCa tissue, namely, secreted protein acidic and rich in cysteine, glutathione peroxidase 3 precursor, zinc alpha 2-glycoprotein, cofilin-1, and heat shock protein-90β. When investigated in urine samples, zinc alpha 2-glycoprotein was found to be discriminative in early diagnosis of PCa (82).

A group of researchers led by Wang et al. described the prospectus of urine exosomal proteins as biomarkers and problems faced when validating these MS-based results using clinically relevant techniques such as enzyme-linked immunosorbent assay (ELISA) and Western blotting. The group observed that ELISA analysis of combined flotillin 2 and Parkinsonism associated deglycase (PARK7) had a value of 68% sensitivity and 93% specificity, distinguishing PCa patients with healthy controls. Western blotting analysis for flotillin 2, late endosomal/lysosomal adaptor, MAPK and MTOR activator 1 (LAMTOR1), transmembrane protein 256 (TMEM256), and RAS-associated protein 3B (RAB3B) proteins showed higher expression of these proteins in urinary exosomes of PCa patients compared to healthy patients (83). Validations in a higher number of patient samples may improve their utility as potential biomarkers in clinical settings.

Proteomic-based discovery of biomarker does not always translate to a diagnostic utility. Validation of biomarkers in a large pool of patient samples is an important step after the discovery phase studies have been conducted. The primary challenge in targeted proteomics approach for biomarker discovery and validation is arduous assay optimization. A prevalidation workflow was developed by Adeola et al. to clinically validate biomarker discovery. This bioinformatics verification platform used IHC and SRM verification as a preliminary step. The proteotypic peptides successful in this step were validated by PRM. The results revealed 12 potential biomarkers differentiating PCa with healthy control samples (84). This pipeline serves as better validation tools compared to Western blotting and IHC analyses.

**Figure 2:**
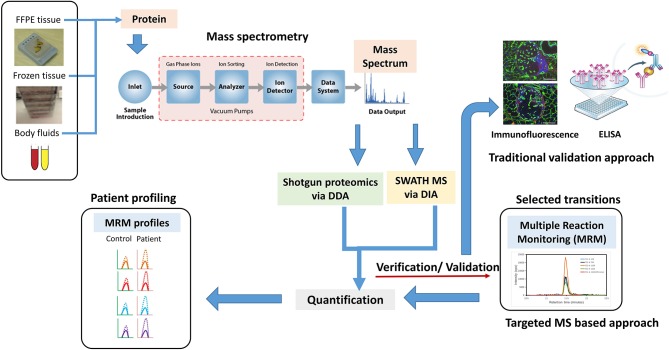
A new age proteomic approach for better validation for biomarker for prostate cancer (PCa) to improve diagnosis and prognosis. A shotgun proteomic approach via data-dependent acquisition (DDA) in combination with sequential window acquisition of all theoretical fragment–ion spectra (SWATH)–MS via data-independent acquisition (DIA), integrative proteomics is a better proposition in identifying biomarkers and protein signatures for improving PCa diagnosis, managing disease, and predicting treatment outcomes. Traditional approaches like immunofluorescence, Western blotting, and ELISA can be replaced by MS-based targeted approaches such as multiple reaction monitoring (MRM) to detect, distinguish, and quantify specific biomarkers obtained from specific samples sets. MRM profile image adapted from Arora et al. (85).

Similarly, shotgun proteomics via data-dependent acquisition (DDA) has assisted in MS detection, but the problem of missing data with data acquisition has haunted the area of discovery proteomics (Figure 2). Several data processing tools and spectral libraries are being developed to overcome this problem (86). Sequential window acquisition of all theoretical fragment–ion spectra–MS is now being used in many research labs for biomarker discovery wherein the combination of DDA and data-independent acquisition (DIA) is being applied to construct spectral libraries, giving a broad coverage of all precursor ions within a particular *m*/*z* range (87). This precise and reproducible quantification pipeline shows promise in PCa biomarker discovery in the future. In a recent study using integrative proteomics in PCa, sequential window acquisition of all theoretical fragment–ion spectra–MS was used to identify novel molecular pathways with the proteome of BPH, PCa, and CRPC patients. The proteomic data identified changes in tricarboxylic acid cycle, which was comparatively altered in CRPC vs. PCa and PCa vs. BPH patient samples. More than 3,000 proteins were quantified in these clinical sample batches with importance of proteins in mechanisms supporting PCa growth and progression. These study provided an integrative robust analysis that underlying proteomic changes are not always related to copy number, RNA expression, and DNA methylation (88). With respect to precision medicine for PCa, these high-throughput proteomic technologies will hold greater value in disease management and improved prognosis and diagnosis. In addition, for personalized medicine for PCa, proteomic signatures will be more helpful in achieving the ambitious goal of diagnosis and treatment outcome prediction. Technological advances in this field have led to development of new methods to screen and generate multiple candidate protein biomarkers with the help of LC-MS/MS. Multiple research has identified various candidate biomarkers, but a greater concern still holds on the verification and validation of these selective biomarkers. MRM approach can be used to develop assays providing absolute quantification of protein biomarkers in clinical samples. Using this method, Fortin et al. showed clinical quantitation of PSA with concentrations from 4 to 40 ng/ml showing good correlation with ELISA tests (89). Furthermore, this technique should be used in new protein biomarkers identified in large cohort of patients to validate the efficacy of the biomarkers to be used in clinical settings. In addition, the use of protein biomarker panels needs to be explored more using MS-based techniques, which could lead to increased sensitivity and specificity. This kind of pattern studies have now been used in several publications, but the pretext of proteomics in discovery and verification of these panels needs to be fulfilled in personalized medicine for the prediction of patients' response to therapy, diagnostic, and prognostic purposes.

## Genome Editing For Precision Therapy

Efforts to treat genetic diseases have always been an area of development and contextualization. Gene editing, redefined and rediscovered, provides an innovative approach to increase gene correction and develop a precision therapy transforming clinical technologies. There are some genetic tools existent for this objective, comprising of zinc-finger nuclease, transcription activation-like element nuclease, and the latest, CRISPR/CRISPR-associated (Cas) system. The latter is more cutting edge due to ease in generation and immense efficacy for gene targeting, engaged to effectively accomplish knockouts in different human cell lines (90). Proficiency, precision, and high-throughput screening demonstrate that CRISPR/Cas9 editing technique can be contemplated for therapeutic applications. The first CRISPR phase I clinical trial started in 2016 in China, wherein CRISPR/Cas9 is performed *ex vivo* to knockout programmed cell death protein-1 in T cells (NCT02793856). These engineered cells have been selected and infused into patients. Similar clinical trials targeting programmed cell death protein-1 on PCa (NCT02867345), renal cell cancer (NCT02867332), bladder (NCT02863913), and esophageal cancer (NCT03081715) have also been started.

Genome-editing technology targeting chromosomal breakpoints could provide an alternative methodology treating human cancers via inhibiting fusion genes. Herpes simplex virus type 1 thymidine kinase (HSV1-tk) is a prodrug converting enzyme that forms thymidine monophosphate by phosphorylating thymidine, an active ingredient in DNA synthesis mechanism. HSV1-tk, unlike its mammalian equivalent, also phosphorylates ganciclovir (prodrug), a synthetic nucleoside homolog (91). The phosphorylation step leads to elevated ganciclovir monophosphate levels in mammalian cells. This monophosphate is modified to triphosphate, which blocks DNA synthesis. Cells are immune to this phenomenon lacking HSV1-tk due to failure in phosphorylating ganciclovir. Chen et al. using a mutated Cas9 used CRISPR to insert HSV1-tk in the chromosomal breakpoints of *TMEM135*–*CCDC67* and *MAN2A1*–*FER* fusion genes. This led to cell mortality in cell culture system and reduction in tumor mass and mortality in mice grafted with human prostate and liver cancers (92). The research did not observe any major cytotoxicity in cell or animal models. The authors discussed a castrate-resistant improved methodology of genome editing with tumor immunotherapy and other therapeutic treatments when obligatory for improved therapeutics. Although this study reduced the cytotoxicity, a major issue concerning CRISPR models has been non-specificity. The “unknown” stress induced by CRISPR on the genome via development of new polymorphisms could lead to consequences not intended and ultimately producing a negative effect. The size of Cas9 protein has always been an issue that has resulted in unintended editing in the genome. New Cas proteins have been identified, which are smaller in size. In a recently published study in *Nature*, a group of researchers from University of California have revealed a distinct genome editing platform and termed as CRISPR-CasX system (93). The authors, through cryo-electron microscopy, elucidated the structure of CasX in its assembly state determining its small size and DNA cleavage properties. This protein is derived from non-pathogenic microbes found in groundwater and sediment. The additional property of non-immunogenicity makes it an ideal genome editing tool compared to Cas9 and Cas12a. Once this system gets developed in humans, it would provide opportunities for therapeutic delivery, and the notion of safety can be put to rest.

GPRC6A, a G-protein-coupled receptor, has been found to be a sensing receptor promoting the progression of PCa and a target for developing antagonists to treat PCa. Ye et al. used CRISPR-Cas9 technology to delete *GPRC6A* in human PC3 cells and observed a diminishing reaction to L-Arg, osteocalcin, and testosterone stimulation of ERK, Akt, and mTOR phosphorylation with reduced cell proliferation and downregulation of *OCN, PSA, MMP9, BMP3, RUNX2*, and *VEGF* genes, which are involved in PCa progression (94). This study indicated that *GPRC6A* editing could reduce expression of enzymes regulating intratumor androgen biosynthesis. Additional efforts to develop antagonist against this target could lead to development of new treatments for PCa. A focal limiting feature in the development of CRISPR in clinical trials is the selection of the delivery method. Developing a delivery system for therapeutic targeting via CRISPR/Cas9 requires specific administration and formulation of the delivery reagent. Zhen et al. established an aptamer-liposome centered CRISPR/Cas9 chimera, which recognizes prostate-specific membrane antigen (PSMA) receptor expressed on PCa cells. The team designed chimeras with RNA Aptamer A10, which have the capability to bind to PSMA. The research suggested A10-liposome-CRISPR/Cas9 chimera delivery system as an appropriate method targeting tumors *in vivo*, exonerating its usage as a viable therapeutic treatment method for human PCa (95). Evaluation of delivery methods and potential genotoxic effects of using CRISPR can only be done in an efficient manner when additional preclinical studies are implemented. There is no guarantee of therapeutic efficacy even after developing new delivery methods of CRISPR vectors. In the coming time, whether CRISPR makes a difference in PCa or not is up for debate but is definitely worth the effort.

## Non-coding-RNA-based Diagnostics And Therapeutics

Non-coding RNAs (ncRNAs) are generally distributed into dual primary groups in view of their sizes: small ncRNAs (<200 bp) and long ncRNAs (lncRNA, >200 bp) (96). Small ncRNAs primarily comprise of circular RNAs (circRNAs), microRNA (miRNA), small nuclear RNA (snRNA), and small interfering RNA (siRNA), besides transfer RNA (tRNA), and ribosomal RNA (rRNA) (97). LncRNAs are divided into inter- and intragenic lncRNAs vis-à-vis their positions in the genome comparative to protein coding genes (98); intragenic lncRNAs can be additionally categorized as exonic, intronic, and overlapping lncRNAs (98). Non-coding RNAs such as lncRNAs, miRNAs, and circRNAs have demonstrated lineage-specific patterns, which could be used as biomarkers to discriminate between different tissue types (99). This section will discuss the different preclinical and clinical aspects of ncRNAs like miRNAs, circRNAs, and lncRNAs, which have been well researcherd in PCa.

**Figure 3:**
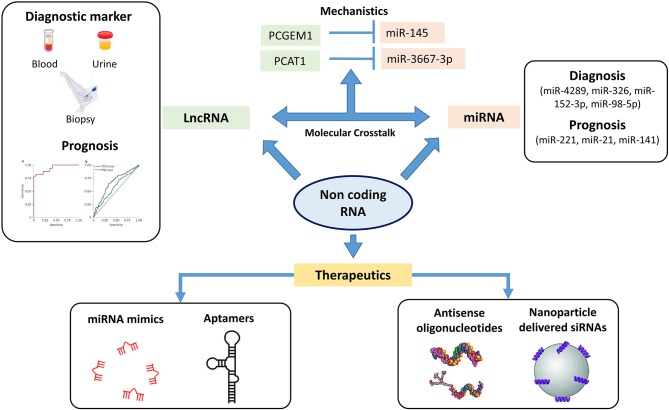
Non-coding genome plays an important role in oncogenesis. The figure highlights the different sets of non-coding RNA which can be used therapeutically as well for diagnosis and prognosis. MicroRNA (miRNA) profiles have been screened in identifying differences in localized and metastatic form of prostate cancer (PCa) with studies also targeting these miRNAs as therapeutics. Long non-coding RNA (lncRNA) including PCA3 and second chromosome locus associated with prostate-1 (SChLAP1) have been screened as potential prognostic markers wherein a PCA3 test has already been FDA recognized as urinary diagnostic biomarker. The crosstalk between these lncRNAs and miRNAs have been studied for identifying novel mechanisms in PCa pathogenesis. The figure highlights the crosstalk between lncRNA PCGEM1 and PCAT1 with miR-145 and miR-3367-3p. ASO and nanoparticle delivered siRNAs based therapeutics have entered the clinical trial stages wherein these non-coding RNAs have been targeted with small antisense oligos to inhibit/restore their activity (⊣ Inhibition).

MicroRNA plays an important role in intracellular processes regulating gene expression at the level of transcription binding to regulatory elements. Several miRNAs have been screened for potential biomarkers (Figure 3) for disease aggressiveness and therapeutic resistance, as well as therapeutic targets (100). miRNA profiles have been studied to identify differences between BPH, localized, and metastatic form of PCa. In a study by Lichner et al., miRNA downregulation was observed in higher Gleason grade prostate tumors compared to lower Gleason grade tumors (101). The same group pointed out that lower levels of miR-331-3p and miR-152 in patients were associated with increased risk of biochemical failure (102). A recent study analyzed circulating microRNAs in patient serum and correlated them with clinicopathological characteristics (103). The authors identified miR-375, miR-106b, miR-21, and miR-141-3p to have increased expression in PCa patients compared to healthy controls. This study discussed the use of miR-375, miR-141-3p, and miR-21 as potential biomarkers, with significant improvement in prediction of PCa in patients. In a review by Matin et al., the diagnostic and prognostic potential of miRNAs in PCa were briefly reviewed wherein different aspects of miRNA expression pattern in tumor tissue and circulating miRNA were discussed in length. The authors also deliberated on the polymorphic aspect of miRNA and how these polymorphisms can predict an individual's response to miRNA therapy (104). Although these findings may shed light on the potential of miRNA as diagnostic, prognostic markers and its therapeutic potential in PCa, reproducibility and validations in large cohorts limit their usage as biomarkers for PCa. Another area of deliberation is the detection of circulating miRNAs in cancer. miRNA detection has been possible by quantitative reverse transcription PCR, next-generation sequencing, microarray, and biosensor-based assays (105–107). Although these strategies have improved detection of miRNAs, an area of contention remains in low abundance of these molecules in serum or plasma and the cost of using these technologies in a clinical setting. Despite various obstacles, targeting miRNA function has been achieved, which gives a clear indication of targeting these molecules for PCa precision medicine.

The purpose of expediting miRNAs as targets for anticancer therapeutics is their dysregulation in different cancers targeting several genes and their capacity to modify phenotype (108). Various strategies ranging from using CRISPR, miRNA sponges, antisense oligonucleotides to small molecule inhibitors have been used to target miRNA expression (104). High-throughput sequencing has revealed miRNA changes in PCa patients, correlating its diagnostic value as a therapeutic target (109). Antisense oligonucleotide (ASO) based therapeutics have been tested in clinics for several years along with RNA-based therapies. Eight of these therapies have reached phase III clinical trials until 2016 (109). miR-16 has been found to inhibit EMT by regulating *p-FAk* and *p-Akt* expression, inhibiting the transcriptional efficiency of *NF-kB* and Slug transcription factors (110). In one of the phase I trials, miR mimetics were used to restore miR-16 (TargomiRs) activity. In a study at Wisconsin Medical College, blood samples were collected at the time of ADT, posttreatment, and upon tumor progression to identify miRNAs and define changes in disease response (ClinicalTrials.gov Identifier: Nbib2366494). The study, once completed, aims to help in identifying specific miRNA changes at different levels of disease treatment for precise decision making affecting patient care. In a clinical phase II study, abiraterone, an androgen synthesis inhibitor, sensitivity to circulating miRNAs are being validated for prognosticating PCa progression (ClinicalTrials.gov Identifier: Nbib1503229). A research study conducted by Liu and group showed miR-34a via repressing CD44 reduced prostate tumor and metastasis in mice (111). This study was the basis of the first miRNA-based therapeutic trial, MXR34 (Nbib1829971), in men delivering the miRNA using liposome nanoparticles (41). This study was conducted in liver cancer patients as the nanoparticles containing the RNA accumulated in liver tissues. These studies could be used to further change the delivery method for prostate-specific targeting. All these clinical studies on miRNA in PCa could lead to a panel of miRNA biomarkers in addition to imaging techniques for predicting disease stage and burden. If these stable miRNAs could predict the primary or metastatic stages of PCa, it would lead to better treatment usage like chemotherapy and androgen-deprivation therapy altering the overall survival of patients.

CircRNAs were primarily studied in plants infected with viruses and subsequently confirmed in animal cells and fungal yeast (112, 113). These RNAs are stable and expressed in saliva, blood, and other body fluids (114, 115). However, these circRNAs have now been studied in PCa tumorigenesis and progressions and their mechanism investigated. To determine the potential of circRNAs as biomarkers, Xia et al. analyzed their expression profiles in prostate tumor and precancerous tissues (116). One thousand twenty-one differentially expressed circRNAs were found in the PCa tumor. The results found two circRNAs—circ_0057558 and circ_0062019—combined with PSA distinguished PCa and BPH patients with increased AUC, sensitivity, and specificity (0.938, 84.5 and 90.9%, respectively) compared to PSA alone (AUC of serum PSA −0.854). Another circRNA, circRNA myosin light chain kinase was found to be upregulated in PCa tissues and cells, increasing cell proliferation, invasiveness, and migration (117). The levels of circRNA-MYK were found to be affected by miR-29a underlying the sponge mechanism of these RNAs. miR-29a expression was found to be lower in tumor samples compared to normal tissues, negatively correlating with the expression of circRNA–myosin light chain kinase. Recently, a study identified hsa_circ_0004870 to be associated with enzalutamide resistance in PCa, highlighting critical roles of these RNAs in developing resistance in CRPC (118). Likewise, circ-102004 expression was also found to be higher in PCa tissues having an oncogenic role in stimulating cancer cell migration and invasion (119). Emerging interests in developing these circRNAs for diagnostic and therapeutic purposes have resulted in several databases being produced like CircBase, CIRCpedia, and CSCD to help understand the mechanism and potential of these RNAs (99). A study by Vo et al. discovered circAURKA as a potential marker for neuroendocrine PCa. RNA-seq on 144 localized prostate tumors by Chen et al. identified circCSNK1G3 interacting with miR-181 and promoting cell growth (120). Taken together, all these studies and the emerging circRNA databases have advanced the knowledge of exploring these RNAs as new targets. In addition, some of these studies also highlight their importance in developing novel biomarkers at different stages of PCa.

Recent studies have revealed lncRNAs like prostate cancer antigen 3 (*PCA3*), second chromosome locus associated with prostate-1 (SChLAP1), and PCa associated non-coding RNA transcripts (*PCAT*s) as potential prognostic biomarkers. *PCA3* is found to be upregulated in prostate tumors in comparison to non-tumor cells (121). PCA3 can be identified in urine, and the PROGENSA *PCA3* test is the primary urine-based molecular diagnostic test sanctioned by the FDA (122). In addition, meta-analysis of several studies established the validity of urine *PCA3* levels for PCa diagnosis, with a sensitivity of 62% and a selectivity of 75%. In the receiver operating characteristic curve (ROC), this outcome interpreted as an AUC of 0.75, promoting PCA3 as a sensible marker for PCa diagnosis (123). Comparable outcomes were acquired in a subsequent independent meta-analysis wherein the sensitivity and selectivity for PCa diagnosis were 57 and 71%, respectively; correspondingly, the AUC was 0.711 (124). As its plasma expression levels draw a parallel with tumor aggressiveness, as categorized by the Gleason score, circulating PCA3 can also reveal the aggressiveness of PCa (125). Although *PCA3* improves the specificity for PCa detection in comparison to serum PSA, it is not sufficient to use it alone in making a decision for initial biopsy due to its lower sensitivity (125). Multiparametric magnetic resonance imaging (mpMRI) is a technique increasingly being used for prostate tumor detection and localization with high sensitivity and high-negative predictive value. Biomarkers like *PCA3* in combination with mpMRI could predict high grade PCa more suitably. An observational study led by Hendriks and colleagues investigated association among a novel biomarker-based risk score (SelectMDx, MDxHealth, Irvine, CA), mpMRI outcomes, and biopsy Gleason score (126). The novel SelectMDx risk score surpassed *PSA* and *PCA3* in the ROC curve analysis with an AUC of 0.83 vs. 0.66 and 0.65. The study suggested using this novel risk score to identify PCa risk patients for advanced diagnostics, reducing overtreatment and avoidable diagnosis. Recently, Sanda et al. argued on collective urine based evaluation of *PCA3* and *TMPRSS:ERG* fusion after DRE to improve specificity for aggressive PCa and for fending needless biopsies, which could prevent increased healthcare costs (127). Van Neste et al. evaluated a urine-based molecular marker risk score to aid in detection of high-grade PCa. This messenger RNA (mRNA) test combined molecular profiling data with clinical risk factors to precisely identify patients with high-grade PCa. mRNA expression levels of *HOXC6* and *DLX1* combined with *PSA*, age, DRE, prostate biopsy, and family history were used to detect high-grade PCa accurately with the researchers claiming that, clinically, it could reduce biopsies and overtreatment (128). Another group examined the ratio of Hepsin mRNA to *PCA3* with serum PSA to improve prediction of PCa status. The results showed the ratio of Hepsin:*PCA3* with serum PSA as a better predictor than PSA alone of PCa status and risk (129). These results are still not sufficient enough to be recommended as stand-alone tests for identifying PCa; however, these do provide a basis for adding other markers to improve patient diagnosis and selecting patients for biopsies and treatment.

Many lncRNAs also play crucial roles in cancer, from acting as oncogenes or tumor suppressors to regulating oncogenes and tumor suppressors at transcriptional and posttranscriptional levels (130). The idea of targeting lncRNA has been explored by researchers worldwide. In a study by Kanduri et al., the function of lncRNA, *SCAT7*, was blocked using ASO to reduce lung tumors in mice (131). These ASOs were observed to reduce lung tumors by 40–50% when injected twice a week and in 15 days. *ARLNC1*, an lncRNA, was found as a novel regulator of AR signaling, highlighting its potential as therapeutic target for advanced PCa (132). The application of ASOs has made possible of them being used to target the so-called “undruggable” genome like the lncRNA at the transcript level in PCa treatment. Understanding the molecular mechanism of lncRNA in PCa pathogenesis could yield a new “lncRNA therapy” being developed.

RNA interference (RNAi) using siRNA to treat disease have been the most effective method to date with the first ever drug approved by FDA treating hereditary transthyretin-mediated amyloidosis already in the market. This double-stranded RNA molecule could also become an effective cancer therapy. Various clinical trials are at an advanced stage using this molecule for treating cancer (133). siRNA-based approach has also gained momentum for treating PCa. Recently, nanoparticle-conjugated siRNA targeting PSMA showed significant inhibition of PCa tumor growth *in vivo* (134). Similar results were obtained in mice using an siRNA conjugated with squalene, a non-ionic lipid, targeting *TMPRSS2–ERG* junction oncogene (135). Other molecules like aptamers, which are single-stranded RNA or DNA oligonucleotides, have also been explored as agonists, antagonists, as well as effective drug conjugates (136). OX40, an agonistic aptamer targeting CD134 and 4-1BB, activated T cells and showed antitumor effects (137, 138). This aptamer targeting PSMA has also shown to reduce side effects. Additional research using this molecule has also led to developing an aptamer–antibody complex, called as oligobody, which reduced tumor burden in mice (139). Similar results using aptamer–drug conjugates were obtained in PCa cells, without causing harm to normal tissues (140). These kinds of studies indeed highlight the use of aptamer-based therapies as safe options to treat PCa.

All these types of ncRNAs do provide a novel “out-of-the-box” methods to target PCa; however, precision medicine does need approaches for efficient delivery of these RNAs. The complex RNA-based therapeutics including miRNA mimics and siRNAs do need to overcome the problem of half-life and increased stability to be used in clinics. In addition, miRNAs and lncRNAs have been suggested to be useful biomarkers for monitoring treatment outcomes and responses, although all of them are still in preclinical phases. Only a few of them exist in body fluids, enabling a non-invasive liquid biopsy approach. The short ncRNAs like miRNA and siRNA have been advantageous for safe circulation and uptake, improving bioavailability, which has seen rapid development of clinical trials using these RNAs. Going forward, modifications including nanoparticles and alterations in ncRNA structure could help in overcoming toxicity and reducing off-target effects. Despite these challenges, ncRNA-based therapeutics are envisage to be a powerful treatment strategy for cancer treatment.

## Liquid Biopsy

Tumor heterogeneity has made assessing variations in solid tumors extremely difficult, undermining effective treatment. Disadvantages of tissue biopsies include risk of infection and prolonged recovery time for patients, insufficient tissue for histological and molecular analysis of patients, and rebiopsy for additional genotyping and molecular profiling (141). PSA levels as an invasive test has failed to monitor disease burden and identify impact of therapies, reflecting its inefficiency as a dominant marker for PCa monitoring. Looking into the limitations of traditional biopsy, liquid biopsy gives an alternative approach as an advancing diagnostic tool. The limiting factors for improved treatment decisions—clonal variations and heterogeneity—are better evaluated while analyzing circulating components of blood. Furthermore, the genetic profile of patient tumor subclones is better reflected by liquid biopsy rather than tissue biopsies (142). The potential clinical utility of molecules identified in liquid biopsy includes cell free DNA (cfDNA), circulating tumor cells (CTCs), cell free proteins, exosomes, miRNAs, lncRNAs, mRNAs, and peptides (143). CTCs along with circulating tumor DNA (ctDNA) have been explored to ascertain alterations in the genome in PCa and trace the differential genomic landscape over a period of time (14).

With broader applications of next-generation sequencing technology, the use of ctDNA in screening genetic lesions have become highly specific and increasingly sensitive. From expediting early-stage detection to precisely determining tumor progression, prognosis, and assisting targeted therapy, the use of ctDNA in liquid biopsy represents a revolution in cancer diagnosis, prognosis, and treatment (142). New technologies including digital PCR-based methods, personalized analysis of rearranged beads, tagged amplicon deep sequencing, and untargeted ctDNA approaches have been developed to detect mutations and chromosomal rearrangements in ctDNA. The use of digital PCR yields detection of ctDNA in more than 75% of advanced cancer patients, whereas in patients with localized tumors, the range is around 48–73% (144). The use of ctDNA offers a viable approach in clinical settings for patients with less pain and invasiveness compared to a tissue biopsy method. Recently, a team lead by Sonpavde et al. analyzed ctDNA from blood samples of 514 patients suffering from CRPC. The research found linkage between DNA changes and poor clinical outcomes in 163 patients, 46 of which were treated for CRPC before the study. A high number of genetic alterations were found in AR gene, similar to tumor tissue alterations, and were associated with poor treatment outcomes. The data also detected new *AR, MYC*, and *BRCA* alterations following therapy, which could be targeted using agents like immune checkpoint and PARP inhibitors (145).

A secondary approach in liquid biopsy of PCa patients is the examination of CTCs. These cells have their origin from the primary tumor or metastasis and can be detected in blood. Initially thought as a measure of disease aggressiveness, CTCs with emerging detection technologies have been studied and investigated for diagnosis and cancer management. The advantage of CTCs has been ease of isolation of pure tumor DNA and RNA from single cells for analyzing splice variants, which play a significant role in developing resistance to ADT in men suffering from PCa (146). The development of functional CTC studies has been a challenging task, as only a small number of CTCs can be salvaged from patient blood. To overcome this, new technologies are being developed for CTC culturing. Gao et al. derived 3D organ cultures from patients with advanced PCa, paving the way for the application of CTC cell lines in drug development and targeted therapy (147). Identifying variants of AR via liquid biopsy gives clinicians treating patients with enzalutamide or abiraterone based on resistance and sensitivity. For example, Borgmann et al. checked the potency of darolutamide, a chemically distinct drug compared to enzalutamide and other AR inhibitors. It was shown to have a higher binding affinity to AR, inhibiting cell growth and AR transcriptional activity in enzalutamide-resistant CRPC (148). Based on AR mutational status, probably in CTCs and its sensitivity to darolutamide, it can be used in precision oncology targeting mutated form of AR. CTCs have extensive heterogeneity, showing capacity to expand and form clusters that can traverse narrow capillaries and retain their properties upon reaching wider blood vessels (149). These clusters have been found to metastasize compared to single CTCs, ultimately decreasing overall survival of patients (150). Recently, Gkountela et al. profiled the DNA methylation landscape of single vs. clustered CTCs from breast cancer patients and mouse models to dig deeper into the biological features of CTC clusters (151). These CTC clusters shared several properties with stem cells including regulating self-renewal and proliferation. The study found hypermethylated sites for transcription factors associated with proliferation and stemness in CTC clusters. The researchers also identified NA+/K+ ATPase inhibitors to be enabling in dissociating CTC clusters and remodeling DNA methylation and metastatic suppression. The phenotypic features of CTC clusters and an insight into their biology published in this study provide a rationale for applying compounds for treating breast cancer patients.

Currently, an FDA-approved CTC test by CELLSEARCH® system (Menarini Silicon Biosystems) is being used to detect CTCs in peripheral blood (152). The kit detects CTCs of epithelial origin in whole blood, which helps in monitoring patients with prostate, colorectal, or metastatic breast cancer. CELLSEARCH® CTC test has been argued to be used with other imaging and laboratory tests and physical examination for better prediction. This test relies on a single marker (EpCAM+) for CTC isolation, which has shown limitations. Another test by a German company, The Maintrac® CTC count test, measures cancer stem cells in blood. The CTC count indicates cancer aggressiveness and monitors treatment response. These tests, used every 3–6 months, is the best way to determine the risk of cancer spread and relapse, monitor treatment efficacy, and assess cancer aggressiveness. Many studies have proven the significance of Maintrac® diagnostics and are being employed in the case of almost all solid tumors (153–155). A group of Chinese researchers claimed to develop a new platform, subtraction enrichment and immunostaining-FISH to analyze CTCs for early screening of cancer in healthy people (156). They claimed the subtraction enrichment and immunostaining-FISH platform developed had a higher CTC detection rated than the CELLSEARCH system. The subtraction enrichment of the platform used immunomagnetic particles conjugated with anti-CD45 antibody, which does not depend on EpCAM expression of CTCs that may decrease during EMT. The platform identified aneuploidy CTCs in addition to cytokeratin+ (CK+) CTCs (157).

In summary, these technologies represent a potential tool for early-stage cancer screening as well as detecting aggressiveness and monitoring treatment efficacy relapse, providing a source for the use of liquid biopsy for precision medicine.

## Summary

The belief of precision oncology stems from developing effective therapeutic approaches for individual patients. Identification of new disease pathways and the development of pathway inhibitors have indeed contributed to the decline in PCa mortality in the last decades. Nonetheless, these inhibitors have, over a period of time, led to developing resistance in patients. Henceforth, improvement in developing novel therapeutics is the way forward for treating PCa. Therapeutic interventions in gene fusions targeting ERG and other ETS family members are an important opportunity to derive novel inhibitors that could substitute the current chemotherapies to treat resistant and metastatic disease. From using docetaxel and cabazitaxel in improving survival rate of PCa patients to developing new ASOs, precision medicine in cancer has taken big leap in this decade. A randomized trial of using Custirsen (OGX011), a second-generation ASO inhibiting clusterin, an upregulated protein, during chemotherapy, in combination with cabazitaxel and prednisone in CRPC patients, was conducted in Europe where patients have been recruited from eight countries to check for increased survival after the treatment (ClinicalTrials.gov Identifier: NCT01578655). The results exhibited no survival benefits to metastatic CRPC patients resulting in a failed trial. This kind of trials even after exploratory analysis in the *in vitro* and *in vivo* systems fails to deliver on the promise of a personalized approach to treatment. These trials are still far-fetched from having efficacy; however, use of biomarker-driven strategies and scrutinizing the phase I/II proof of concept studies is the way forward. A recent analysis showed ~75% drugs developed accomplished regulatory authorization with this idea of biomarker-driven studies and positive POC in comparison to 15–30% with no putative biomarker and negative or no POCs (158).

The capacity of proteomics to examine and evaluate human disease has been fascinating, leading to new approaches being developed in this century. Proteogenomics is being discussed worldwide to solve problems in diseases, and this integration of proteomics and genomics presents an important opportunity for clinicians for early intervention and consistent monitoring (159). MS has already revolutionized the fields of microbiology and bacteriology in clinical settings; its full potential in the field of clinical oncology is widely being debated. The increased capacity of MS-based qualitative assays has increased specificity in diagnostics. In spite of all its advantages, drawbacks including data acquisition and reproducibility provide a concern for proteomics to be used in clinics. The answer to these questions could lie in using untargeted DIA having high multiplexing and comprehensive quantification capability of thousands of analytes with high specificity and selectivity. Developing a robust MS system with automatic quality control which can be operated by a non-expert could allow clinicians to accept this technology and gain acceptance in the community.

Genome editing has redefined the scientific field with potential ranging from basic sciences to precision therapy approaches. The genotype-phenotype relationship in human cells can be altered using this strategy where disease models have been tailored to edit genetic variations (160). Although genome editing has been a fancied approach for many scientists, arguments regarding its efficacy and the stress induced by it on whole-genome levels make this revolution a social stigma.

The field of oncology was modernized when the non-coding genome was found to affect disease at different levels. Studies indicated that non-coding RNA could serve in the diagnosis and prognosis of cancer (161). RNAi targeting by miRNA and lncRNA has led to success in laboratory settings with clinical trials with non-coding RNA therapies well underway. The year 2018 was a landmark year for RNAi with FDA approving Alnylam's ONPATTRO (Patisiran), the first drug to use this system to reduce transthyretin expression, which causes transthyretin-mediated amyloidosis in adults (162). Other RNAi therapeutic trials are underway at phase III stages for hemophilia, hypercholesterolemia, and acute hepatic porphyrias (163). These efforts may be defining in generating new treatment modules using RNAi technology to improve precision medicine of cancer patients. Liquid biopsy profiling has opened a new avenue compared to the traditional tissue biopsies in harvesting biomarkers like CTCs CtDNA from body fluids. This approach could help evaluate relapse in patients already undergoing treatment and monitor treatment response, guiding to a more personalized approach to cancer treatment.

This concept of precision medicine propelled by the increasing knowledge of genomics, proteomics, and development of new technologies is still in its infancy. The major challenge in developing an efficient therapy is the heterogeneous and multigenic nature of cancers. The results from various studies worldwide look promising for the development of precision medicine in PCa in the near future. However, there is still more to comprehend before precision treatment of PCa can reach the clinics.

## Author Contributions

All authors listed have made a substantial, direct and intellectual contribution to the work, and approved it for publication.

### Conflict of Interest

The authors declare that the research was conducted in the absence of any commercial or financial relationships that could be construed as a potential conflict of interest.
